# Advances in new psychoactive substances identification: the U.R.I.To.N. Consortium

**DOI:** 10.1080/14756366.2017.1333987

**Published:** 2017-06-20

**Authors:** Elisabetta Bertol, Fabio Vaiano, Francesco Mari, Maria Grazia Di Milia, Silvia Bua, Claudiu T. Supuran, Fabrizio Carta

**Affiliations:** a Forensic Toxicology Division, Department of Health Sciences, University of Florence, Florence, Italy;; b Dipartimento Neurofarba, Sezione di Scienze Farmaceutiche e Nutraceutiche, Polo Scientifico, Università degli Studi di Firenze, Sesto Fiorentino, Florence, Italy;; c Dipartimento di Chimica, Laboratorio di Chimica Bioinorganica, Università degli Studi di Firenze, Sesto Fiorentino, Florence, Italy

**Keywords:** New psychoactive substances, drugs, multi-analytical approach

## Abstract

Identification of new psychoactive substances (NPS) in biological and non-biological samples represents a hard challenge for forensic toxicologists. Their great chemical variety and the speed with which new NPS are synthesised and spread make stringent the need of advanced tools for their detection based on multidisciplinary approaches. For this reason, in August 2016, the “Unit of Research and Innovation in Forensic Toxicology and Neuroscience of Addiction” (U.R.I.To.N.) was founded by the Forensic Toxicology Division of the University of Florence. In this Research Unit, various professionals (i.e. forensic toxicologists, chemists, physicians) collaborate to study all the aspects of drugs of abuse, especially NPS. Herein, we describe the multidisciplinary approach comprising liquid chromatography coupled to tandem mass spectrometry (LC–MS/MS), gas chromatography hyphenated to mass spectrometry (GC–MS) and solution nuclear magnetic resonance analysis that allowed the identification of three NPS such as 1-(benzofuran-5-yl)-*N*-methylpropan-2-amine, 2-amino-1-(4-bromo-2,5-dimethoxyphenyl)ethan-1-one (bk-2C-B), and 3-(2-aminopropyl)indole (α-methyltryptamine) in seized materials.

## Introduction

Recent survey data (2016) show how the drug arena is getting far more complicated compared to years before. As presented in the last European Drug report^1^, not only we assist to the continuous development of “new drugs”, but also to the upwards trending of the more established ones, with the addition of new production sites even in Europe. The term “new drugs”, or “new psychoactive substances” (NPS), indicates a large number of synthetic molecules which belong to a vast array of chemical families. NPS are usually designed on the chemical scaffolds of classical drugs of current use for abuse and/or therapeutic purposes. Since the 1970, many NPS have been synthesised and described. Nowadays, by means of the introduction of known NPS within the illicit drug market together with new ones, there is a reappraisal of many of them^2^. The EU Early Warning System reported in 2014 that almost 5000 (i.e., 40 tonnes) seizures of NPS across European countries were accomplished, and among them NPS containing cathinones and synthetic cannabinoids accounted for about the 80% of the total^3^. Both the amount and diversity of NPS represent a hard challenge for the Law Enforcement Agencies and for the Forensic Laboratories appointed to detect and identify large quantities of such substances. As for the legal interventions, European members follow some useful and strategic planning tools such as: (i) EU policy cycle on organised and serious international crime, (ii) EU security strategies, and (iii) the EU drugs strategy 2013–2020 and its current action plan 2013–2016.

**Figure 1.**
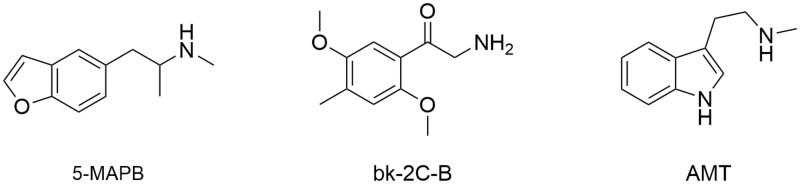
Chemical structures of 5-MAPB, bk-2C-B and AMT.

**Figure 2.**
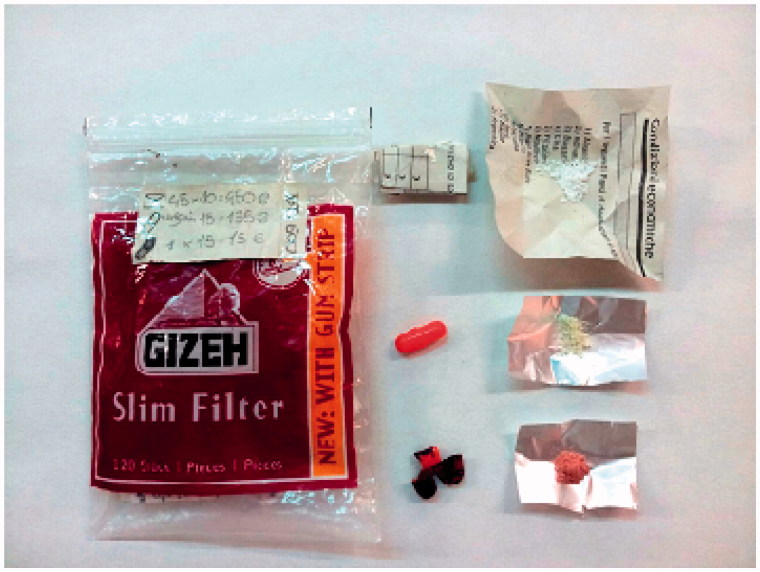
Picture of the seized material.

NPS legal status can differ from country to country since they are not under control of the International Drug Control Conventions^4^. That implies national legislations to be continuously updated on each new molecule and, in order to do so, the first step is to get them identified. Under the Forensic Toxicology perspective, the breadth of the challenge is warring as well. As reported into the UNODC Early Warning Advisory (EWA), 600 NPS were present on the market up to December 2015, and since then probably a larger number has become available^5^. Due to the incessant introduction in the market of new NPS as well as to the lack of reference standards and not yet established effective routine methods for their detection, there are challenges in the identification of some new chemical entities proposed as drugs. Thus, over the last years, several new analytical methods have been developed in order to improve the detection proficiency towards NPS^6^. Most of them require advanced technologies, such as liquid chromatography coupled to tandem (LC–MS/MS) or high resolution mass spectrometry (LC–HRMS). These are very performing tools but could be not enough when considering a totally unknown compound or a reference material is unavailable. In these cases, uncorrelated and more specific chemical analysis is surely beneficial^7^. The nuclear magnetic resonance (NMR) technique can provide detailed information when a structure identification of unknown molecules is required. Considering the urgency of a multidisciplinary approach and the previous NPS detection experiences^8^, our Forensic Toxicology Division established a “Unit of Research and Innovation in Forensic Toxicology and Neuroscience of Addiction” (U.R.I.To.N.) in August 2016. It is the first highly specialised unit in Italy, entirely focused on all aspects of drugs of abuse (NPS on top) by means of a multidisciplinary approach. In U.R.I.To.N., forensic and clinical toxicologists, neuroscientists, chemists, and physicians collaborate with the final intent to obtain a full comprehension of the various aspects related to NSP, which span from the analytical issues to the biological effects and harmful effects. In this paper, we describe the analytical procedure, adopted by our Research Unit on a seized material, which led to the identification of three NPS whose reference standards were not in our possession. 1-(Benzofuran-5-yl)-*N*-methylpropan-2-amine (5-MAPB), 2-amino-1-(4-bromo-2,5-dimethoxyphenyl)ethan-1-one (bk-2C-B), and 3-(2-aminopropyl)indole (α-methyltryptamine, AMT) were identified in three different coloured powders of the same seizure (Figures 1 and 2).

The analytical procedures included a first analysis by gas chromatography with flame ionisation detector (GC-FID) followed by GC–MS and LC–MS/MS detection, all of them performed in the laboratory of our Forensic Toxicology Division. In a later stage, a NMR characterisation was carried out at the Neurofarba Department of the University of Florence.

## Materials and methods

### Seized material

The seized material consisted in a small plastic bag containing: 19 folded slips of paper, 4 orange capsules, and 1 orange capsule coloured in blue. A white amorphous powder was found in all the slips of paper and in three orange capsules, a light yellow amorphous powder in the fourth orange capsule and a grey/red amorphous powder in the blue coloured capsule. Upon the plastic bag were listed the prices of each dose (Figure 2).

### Chemicals and reagents

LC–MS CHROMASOLV^®^ methanol (MeOH), LC–MS CHROMASOLV^®^ acetonitrile (ACN), LC–MS CHROMASOLV^®^ water (H_2_O), dimethyl sulfoxide-d_6_ (DMSO-d_6_) “100%”, 99.96 atom %D, and heavy water (D_2_O) 99.9 atom % D were purchased by Sigma-Aldrich (St. Louis, MO).

### Sample preparation

GC-FID, GC–MS and LC–MS/MS: 5 mg of each sample was added with MeOH (10 mL) and then further diluted to 2 ng/µL.

NMR: 3 mg of each sample was introduced into a 5.0-mm diameter NMR-tube and dissolved with 0.5 mL of DMSO-d_6_.

### GC-FID

The first qualitative analysis was carried out with an Agilent 7890B GC system (Agilent Technologies, Palo Alto, CA) equipped with a FID. The column was an Agilent HP-5 (30 m × 0.32 mm, 0.25 µm film thickness). The starting temperature was set at 100 °C for 1 min, programmed to 125 °C at 25 °C/min for 1 min and to 180 °C at 15 °C/min for 2 min. We routinely use this method to identify the presence of cocaine, heroin, morphine, Δ^9^-tetrahydrocannabinol, ketamine, amphetamine, methamphetamine, 3,4-methylenedioxy-methamphetamine (MDMA), 3,4-methylenedioxyamphetamine, 3,4-methylenedioxy-*N*-ethylamphetamine.

### GC–MS

The GC–MS instrument consisted in an Agilent 7890 A GC system equipped with an Agilent 7683B series autosampler and interfaced to a single quadrupole Agilent 5975C mass spectrometer. The column was an Agilent HP-5MS (30 m × 0.25 mm, 0.25 µm film thickness). The gas carrier (He) flow was constant at 1 mL/min. The samples were analysed in full scan mode (adopted libraries: NIST08, WILEY27, SWGDRUG4). The oven temperature was initially set at 100 °C for 2.25 min, programmed to 180 °C at 40 °C/min and to 300 °C at 10 °C/min for 10 min. Injector and transfer line temperatures were always 300 and 230 °C, respectively. The injection volume was 1 mL in splitless mode. Data acquisition and elaboration were performed using the Agilent MassHunter Workstation software package.

### LC–MS/MS

Analysis was conducted using an HPLC Agilent 1290 Infinity system interfaced with an Agilent 6460 Triple Quad LC/MS (QQQ), equipped with an electrospray ion source (ESI) operating in positive mode. The ESI configuration was: gas temperature 325 °C; gas flow rate 10 L/min; nebuliser 20 psi; capillary 4000 V. Chromatographic separation was performed through a Zorbax Eclipse Plus C18 (2.1 mm × 50 mm, 1.8 m, Agilent Technologies). The mobile phase initially consisted of 5 mM aqueous formic acid (A) and ACN (B) 99:1. Gradient of elution was carried out by increasing the % ACN to 30% within 6 min; to 50% within 2 min; to 100% within 4 min and isocratic for 3 min. The flow rate was 0.4 mL/min until 8 min, then increase at 0.6 mL/min within 2 min. Analysis was carried out first in scan mode (50–500 *m/z*) in positive and negative ionisation and then the collision-induced dissociations (CIDs) were studied at different collision energies (CE, 10, 20, 30 and 40 eV). Agilent MassHunter Workstation software package was used for data acquisition and elaboration.

### NMR

Nuclear magnetic resonance (^1^H NMR, ^13^C NMR) spectra were recorded using a Bruker Avance III 400 MHz spectrometer (Milan, Italy) in DMSO-d_6_. Chemical shifts are reported in parts per million (ppm) and the coupling constants (*J*) are expressed in Hertz (Hz). Splitting patterns are designated as follows: s, singlet; d, doublet; t, triplet; q, quadruplet; m, multiplet; brs, broad singlet; dd, double of doublets. The assignment of exchangeable protons (O*H* and N*H*) was confirmed by the addition of D_2_O.

## Results and discussion

### White powders

**Figure 3.**
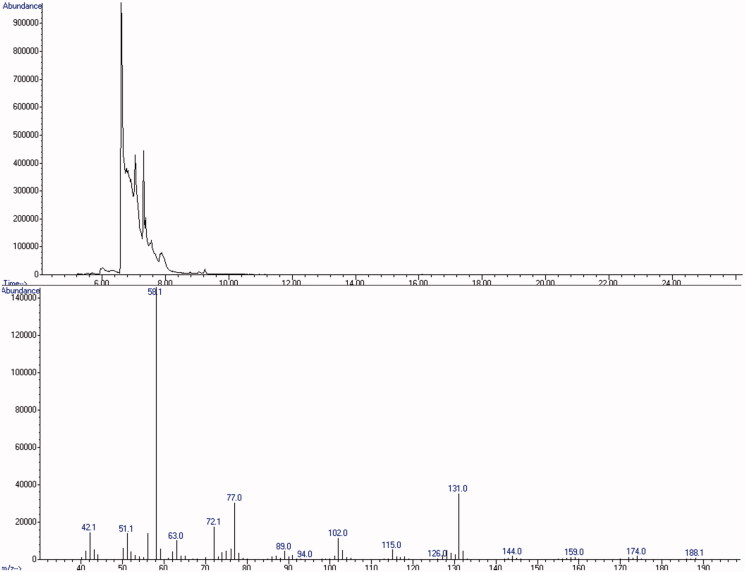
GC–MS chromatogram of the white powder and mass spectrum of 5-MAPB.

**Figure 4.**
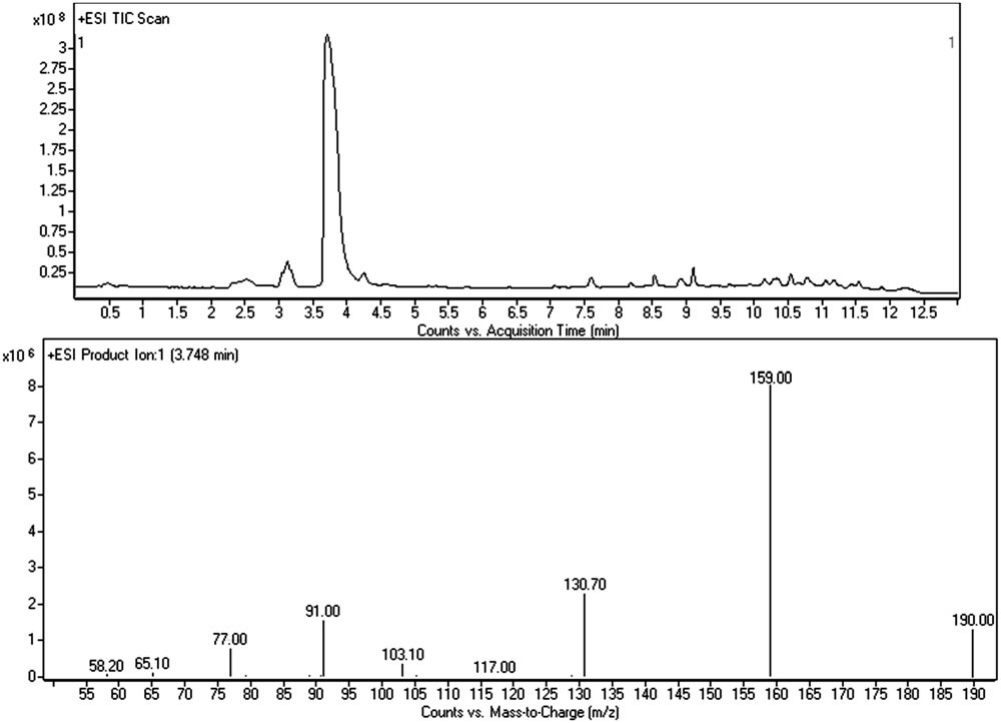
LC–MS/MS chromatogram of the white powder and merged mass spectrum (CE: 10 and 20 eV) of 5-MAPB.

Samples of each white powder were analysed following our protocol. GC-FID analysis excluded the presence of the most common drugs of abuse. GC–MS screening revealed 5-MAPB with a mean estimated concordance among all samples of 72% (peak at 6.793 vs. SWGDRUG 3.0 spectral library, Figure 3). Identification of 5-MAPB was then supported by LC–MS/MS findings (Figure 4) and definitely proved by NMR analysis.

### Light yellow powders

**Figure 5.**
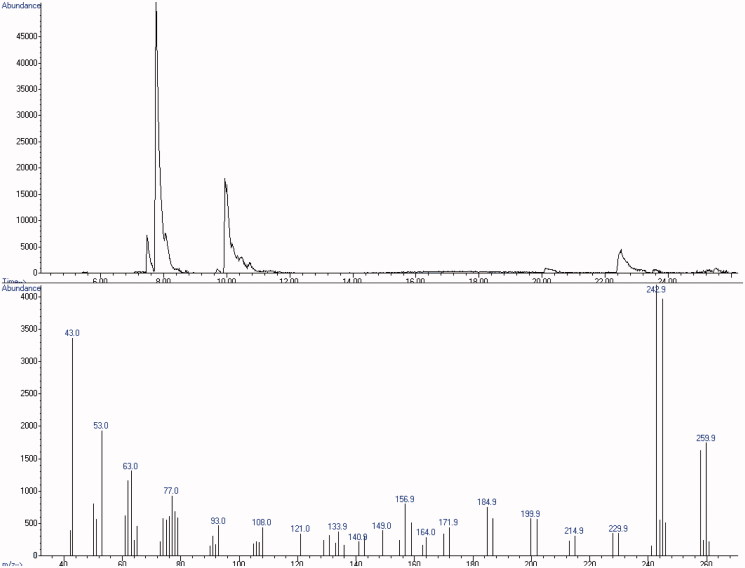
GC–MS chromatogram of the yellow powder and mass spectrum of bk-2C-B.

**Figure 6.**
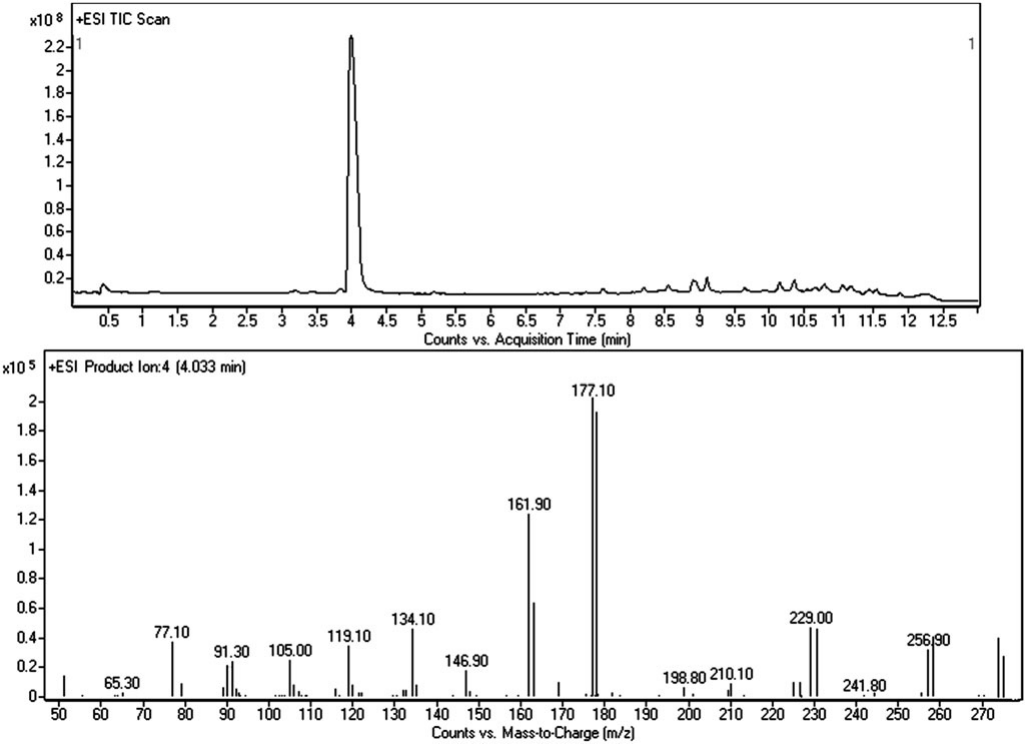
LC–MS/MS chromatogram of the yellow powder and merged mass spectrum (CE: 10 and 20 eV) of bk-2C-B.

Bk-2C-B was identified in all the light-yellow powders. The synthetic cathinone was recognised by the GC–MS screening with a main match of 24% (peak at 7.794 vs. SWGDRUG 3.0, Figure 5). The study of the product ions in LC–MS/MS validated this finding (Figure 6) as the fragmentation profile was highly comparable with the ones reported in literature for bk-2C-B^9^. NMR confirmed this hypothesis.

### Grey/red powder

**Figure 7.**
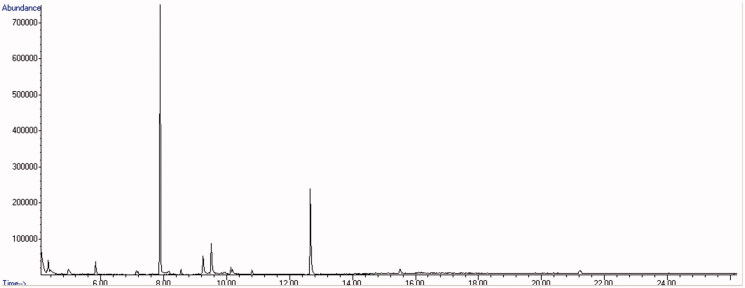
GC–MS chromatogram of the grey/red powder.

**Figure 8.**
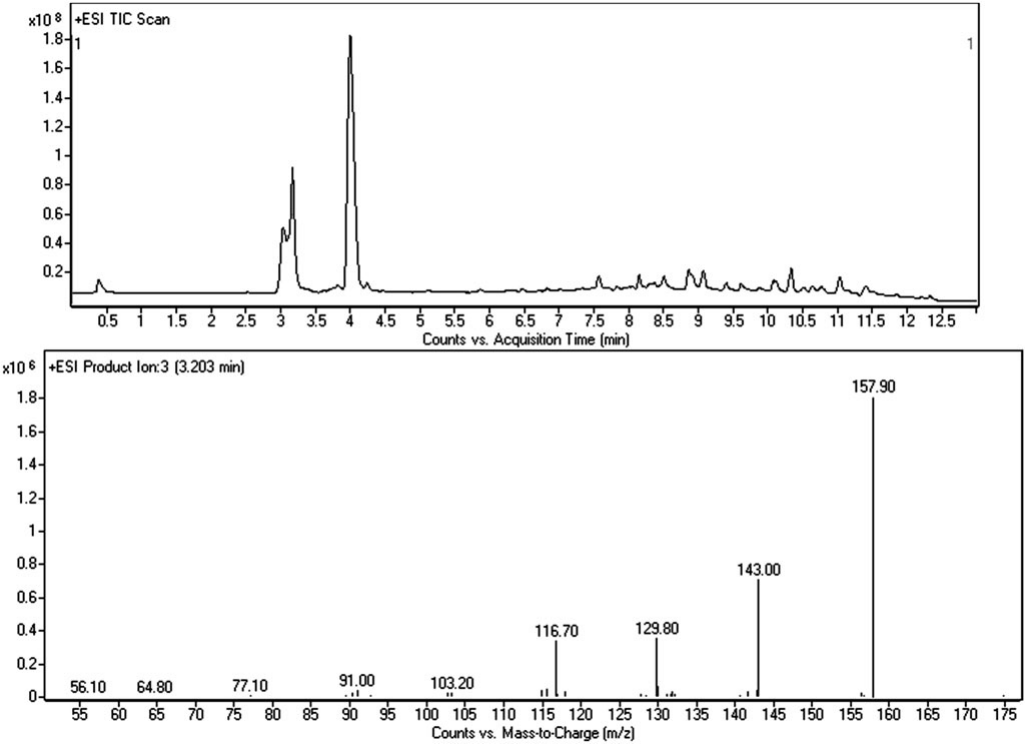
LC–MS/MS chromatogram of the grey/red powder and merged mass spectrum (CE: 10 and 20 eV) of AMT.

This powder was not pure, but consisted in a mixture of the previous substances and a third compound whose actual identification was possible by NMR analysis only. GC–MS screening (Figure 7) gave evidence of the sole presence of bk-2C-B (mean concordance between the spectra: 38%), and neither 5-MAPB nor AMT were identified (even though AMT mass spectrum is filed in the SWGDRUG 3.0 library). Thanks to the previous LC–MS/MS findings, we were able to recognise bk-2C-B and 5-MAPB with the same method, while the other compound stayed unknown. The attempt to understand and draw the structure of the latter (peak at 3.203 min, Figure 8), using data by LC–MS/MS only, was quite challenging. The main issue, after the detection of the [M + H]^+^, was the identification of the chemical formula. A couple of structures were hypothesised (mainly AMT and its isomers), and NMR was used to assess the presence of AMT among the others.

## Discussion

In this paper, we report the NPS multidisciplinary detection procedure adopted by the U.R.I.To.N. Research Unit. This procedure was based on a multi-analytical approach where each instrumentation provided specific findings that, once merged, lead to reliable and indubitable structure identification. This kind of strategy represents the state-of-the-art for non-targeted analysis and various technologies can be used and integrated each other. In recent years, LC–HRMS systems have become the first technological choice in structural investigation protocols as they allow to measure the exact mass (and chemical formula) of an unknown compound and to deeply investigate its fragment residues. Besides the usefulness, such a technique requires its data to be confirmed with uncorrelated methods, such as NMR. In our protocol, GC-FID, GC–MS and LC–MS/MS were used, together with NMR, to identify three NPS (5-MAPB, bk-2C-B and AMT) in the absence of their reference materials (Figure 1).

5-MAPB is a 2-aminopropyl-benzofurane analogue of MDMA, not subjected to control in Italy, and used for its stimulant and entactogenic effects^10^. Sahai et al.^11^ showed that 5-MAPB binds the dopamine transporters and slows down the reuptake of electrically evoked dopamine in the rat accumbens. The same authors also demonstrated that 5-MAPB, in analogy to amphetamine-like substances, is responsible of reverse transportation of dopamine induced euphoria, empathy and psychedelic effects^12^. The toxicity profile of subjects exposed to 5-MAPB is similar to the one resulting from the assumption of MDMA^13^. Similar effects were also observed for other benzofuran containing substances, such as the 5-APB (nor-5-MAPB)^14^.

The bk-2C-B is a compound structurally related to 2 C-B, a serotonergic hallucinogen phenethylamine described for the first time by Shulgin and Carter^15^. To date, little is known about the bk-2C-B induced physiological effects, and useful information of this kind can be only collected from forums on the web^16^. The presence of the 2-amino-1-phenyl-ethanone core does not allow bk-2C-B to be included among the analogues of 2-amino-1-phenyl-1-propanone containing compounds, which are under legal control in Italy.

AMT is a stimulant hallucinogen belonging to the chemical class of unsubstituted synthetic tryptamines^17^. This molecule presents a strong effect on the serotoninergic system due to the high affinity for serotonin transporter and receptors. AMT is also active on the dopaminergic and adrenergic receptors with a similar profile as the one of methamphetamine^18^. AMT induces adverse effects including increase of the blood pressure, tachycardia, nausea, impaired coordination, visual and auditory distortions^19^. There have been deaths related to AMT use, especially in co-consumption with other substances (cocaine, amphetamine, MDMA, and cannabinoids)^20^. AMT is still legal in Italy, whereas its isomer 5-(2-aminopropyl)indole (5-IT) is not.

### GC-FID and GC–MS

The first step consisted in the GC-FID analysis aimed to exclude the presence of the main drugs of abuse, in this case amphetamines and cocaine. GC–MS plays a key role in our protocol as it can give an indication about the substances in the sample. For this reason, our mass spectra libraries are periodically updated. The high diagnostic skill of GC–MS screening is valuable for the analysis of the white and yellow powders. Identification of 5-MAPB and bk-2C-B, even if the concordances were not so high (especially for the phenethylamine), gave us a good starting point. The LC–MS/MS analyses were easier because mainly aimed to the confirmation of GC–MS results. Regarding the grey/red powder, the software recognised nothing but the bk-2C-B (38%) as in the yellow one. None of the other peaks were attributed to 5-MAPB and AMT, later detected by LC–MS/MS and NMR analysis. This may be mainly explained by their low amounts in the seized material. In this case, the GC–MS analysis suggested partially true findings underlining how important a multi-analytical strategy is.

### LC–MS/MS

LC–MS/MS was negatively affected by high in-source fragmentation of all compounds (at standard ESI conditions, see “Experimental” section) which makes identification of [M + H]^+^ hard. For 5-MAPB, this drawback was not so intense and the protonated molecule was easy to see at 190 *m/z*. Main fragments were the benzofuranyl propilium and the benzofuranyl methylium ions at 159 and 131 *m/z*. ESI fragmentation was more intense for bk-2C-B giving several product ions. Even though we were able to recognised the protonated molecule and the typical bromine isotopic pattern with the MH +0 (C_10_H_13_^79^BrNO_3_^+^, 274 *m/z*) and the MH +2 (C_10_H_13_^81^BrNO_3_^+^, 276 *m/z*) at similar intensities. The study of CID at different collision energies provided a fragmentation profile highly comparable with the ones described in literature for this compound^9^.

LC–MS/MS scan analysis of the grey/red powder revealed the presence of bk-2C-B (as indicated by the GC–MS), of 5-MAPB and of a third unidentified compound (peak at 3.203 min, main ion ≈ 158 *m/z*, Figure 8). No peaks were observed in negative ionisation mode. Identification of the unknown molecule was carried out through an *ad hoc* strategy consisting both in the study of its mass spectrum and research on compounds’ databases. The first step was the measurement of the molecular weight (MW) reducing the fragmentor value (at 110, 90, 70 and 40 eV) in order to decrease the in-source fragmentation. We identified the [M + H]^+^ at 175 *m/z* with the highest abundance at 70 eV. Since the QQQ does not allow to measure the accurate mass, it was not possible to find the chemical formula. Studying the deconvoluted spectrum, we collected information about the elemental composition. In particular we excluded the presence of Cl, Br and S by the analysis of the isotopic pattern: 175 *m/z* (100%), MH +0; 176 *m/z* (9.8%), MH +1; 177 *m/z* (0.7%), M + 2. Product ion analysis at various collision energies provided several mass spectra comprising specific fragments useful for the structure drawing: the phenyl cation at 77 *m/z* (C_6_H_5_^+^), the tropylium cation at 91 *m/z* (C_7_H_6_^+^), and the phenylvinyl cation at 103 *m/z* (C_8_H_7_^+^). Another important fragment was the one at 158 *m/z*, originated from the protonated molecule by the loss of ammonia (NH_3_, −17 Da), typical of the primary amine (−NH_2_). It was possible to make some conclusions: (1) the compound had at least one phenyl ring equal to 4 unsaturation degrees; (2) C atoms were ≥8; (3) H atoms were ≥7; (4) I atom was not present because of its mass (MW: 129 Da); (5) since the unknown compound had an even MW, according to the empirical nitrogen rule, N atoms must be even. Resuming all these findings and considering only the most feasible atoms’ ratios, the compound could be described by the generic chemical formula of C_8-15_H_7-20_N_2-4_O_0-2_F_0-1_. The mMass – Open Source Mass Spectrometry Tool software (downloadable at http://www.mmass.org/download/) provided us only four suitable chemical formulas: C_10_H_7_FN_2_, C_9_H_10_N_4_, C_10_H_10_N_2_O, C_11_H_14_N_2_. We typed them in various compounds’ databases^21^, but the number of available structures was very huge. For this reason, we looked inside the NPS database of our Research Unit (available at http://allerta.dronetplus.eu/) and we found a correspondence for the formula C_11_H_14_N_2_ in AMT and its isomers. The fragmentation pattern was consistent with their structures and comparing our mass spectrum with the ones described in literature, we observed a high concordance, especially for AMT^20^a.

### NMR

**Figure 9.**
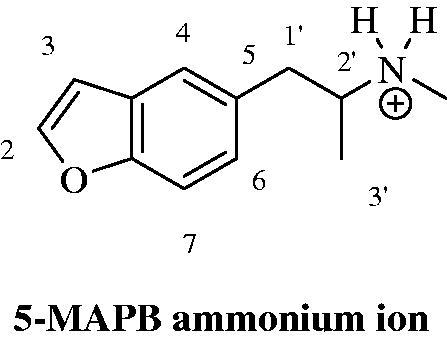
Chemical structure and numbering of 1-(benzofuran-5-yl)-*N*-methylpropan-2-ammonium ion (5-MAPB).

The 400 MHz ^1^H NMR spectrum of the 5-MAPB sample in DMSO-d_6_ (S1 in Supporting information), clearly revealed a doublet signal (d) at 1.15 ppm, with a typical vicinal coupling value of 6.8 Hz integrating for three protons, along with a singlet (s) at 2.16 ppm and integrating for the same value (three protons). These signals can be surely ascribed to 3′-methyl and to the *N*-methyl groups, respectively, placed at the 5-position of the benzofuranic scaffold. The one proton signal at 2′ position was easily identified with the multiplet centred at 3.22 ppm. The enantiotopic methylene protons at 1′ position gave two doublet of doublets (dd) both integrating for one proton and centred at 2.79 ppm (*J* 13.2 and 9.6 Hz) and 3.28 ppm (*J* 13.2 and 4.4 Hz) respectively. As expected each enantiotopic proton gave a geminal (*J* 13.2 Hz) and a vicinal coupling of 13.2 and 4.4 Hz, respectively. Such values are in good agreement with the Karplus equation on the correlation between vicinal coupling constants values and dihedral angles^22^. The presence of the benzofuranyl scaffold substituted at position 5, was assessed by means of: (i) two doublets at 6.95 and 7.97 ppm with a *J* coupling of 2.0 Hz, which can be ascribed to the protons in position 3 and 2 respectively. (ii) The proton in position 6 was identified as the doublet of doublets signal (dd; *J* 8.4 and 1.6 Hz) at 6.95 ppm. The typical vicinal aromatic coupling with the proton at position 7 (8.4 Hz) was easily ascribed, whereas the smaller coupling value of 1.6 Hz can be justified as the results of any rotameric effect given from the closed alkyl side chain and occurring within the NMR experimental acquisition time. (iii) As proof of such interpretation is worth mentioning that the proton at position 4 gave a doublet at 7.65 ppm with a *J* coupling value of 1.6 Hz. (iv) Partially superimposed to the proton signal at position 4, was the proton at 7 position which gave a doublet with a vicinal coupling value of 8.4 Hz. Finally a broad singlet at 8.95 ppm and integrating for two protons was also present. The addition of two drops of D_2_O into the DMSO-d_6_ solution of the sample (S2 in Supporting information) determined a total suppression of such a signal, thus giving evidence that the 5-MAPB is present as an ammonium salt which counter ion was not determined in the set of experiments herein reported (Figure 9).

The ^13^C NMR at 100 MHz afforded 12 signals, and its DEPT-135 deconvolution revealed the presence of one signal down (methylene) and eight signals up, thus in agreement with the 5-MAPB structure (S3 and S4 in Supporting information). A final proof of the NMR based structure elucidation of 5-MAPB was also given from the HSQC experiment which correlated in two dimensions the ^1^H and the ^13^C monodimensional spectra (S5 in Supporting information).

**Figure 10.**
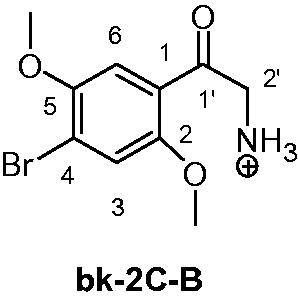
Chemical structure and numbering of 2′-ammonium-1-(4-bromo-2,5-dimethoxyphenyl)ethan-1-one (bk-2C-B).

As for the bk-2C-B sample, the 400 MHz ^1^H NMR in DMSO-d_6_ (S6 in Supporting information) revealed: (i) two singlets at 3.90 and 3.90 ppm, each integrating for three protons which belong to the methoxy moieties present at position 2 and 5; (ii) a singlet at 4.35 ppm and integrating for two protons of the methylene moiety; (iii) two singlets at 7.47 and 7.63 ppm of the aromatic protons at position 3 and 6; (iv) a broad singlet at 8.32 which integrated for three protons. In analogy to the 5-MAPB, the addition of two drops of D_2_O into the DMSO-d_6_ solution of bk-2C-B, determined a total suppression of such a signal (S7 in Supporting information). Therefore, it is reasonable to assume that bk-2C-B is protonated at the nitrogen atom. Again no further experiments were run with the intent to determine which counter ion is associated (Figure 10).

The structure of bk-2C-B was also in agreement with the obtained ^1^H decoupled–^13^C NMR data which afforded for 10 signals, and among them the peak at 192.5 ppm confirmed the presence of the carbonyl at 1′ position (S8 in Supporting information). The DEPT-135 experiment clearly reported four signals up, in agreement with two aromatic CHs and two methoxy groups, and a signal down as only one methylene group was present. Finally, the HSQC experiment determined a clear correlation between the protons and the carbons of the bk-2 C-B claimed structure (S9 and S10 in Supporting information).

The identification of the remaining compound 3-(2-aminopropyl)indole (α-methyltryptamine, AMT) by means of NMR techniques proved to be quite demanding and not feasible, as the substance was present in very small amounts. Attempts to collect a sufficient quantity of material to be used for NMR investigations were done by means of preparative silica gel thin layer chromatography separations, using 20% methanol in dichloromethane as mobile phase. The 400 MHz ^1^H NMR in DMSO-d_6_ of the collected material (not shown) revealed a very small amount of the α-methyltryptamine present. Among the key signals necessary to undoubtedly assign the structure only the peak at 8.00 ppm and 2.52 (overlapped with the residual DMSO) were identified 23.

## Conclusion

The multidisciplinary approach, herein described, well represents an effective attempt to face the NPS detection challenge. The establishment of a highly specialised research unit, “U.R.I.To.N.”, allowed to improve the identification skills of our Forensic Toxicology Division, as well as the exchange of knowledge and know-how among scientists with different expertise. In this paper, we applied this cooperation strategy to the identification of 5-MAPB, bk-2C-B and AMT in three different coloured powders from the same seizure. Their recognition was possible, even without the reference standards, by the combination of advanced analytical techniques (GC–MS, LC–MS/MS, and NMR) that provided peculiar information useful to puzzle up the molecular structures of the NPS present in the seized materials. The advantage offered by the cross interaction between the consortium members is also represented by the reduction of the times associated to the identification of the substances. Typically in a time frame of 4–6 h the identification of multiple NPS as rough substances in seized material is complete. This is an important example of how the collaboration and cooperation between different fields of knowledge is fundamental and desirable in order to be more effective and responsive to the NPS phenomenon and its continuously changing nature.

## Supplementary Material

IENZ_1333987_Supplementary_Material.pdf
