# Supplementary material for: Advances in new psychoactive substances identification: the U.R.I.To.N. Consortium
Source: J Enzyme Inhib Med Chem. 2017 Jun 20;32(1):841–9. doi: 10.1080/14756366.2017.1333987 (PMC6445230; doi:10.1080/14756366.2017.1333987)
Supplement: IENZ_1333987_Supplementary_Material.pdf [file IENZ_A_1333987_SM5160.pdf]

Supporting Informations for

Advances in New Psychoactive Substances Identification: The U.R.I.To.N Consortium

Elisabetta Bertol[a], Fabio Vaiano[a*], Francesco Mari[a], Maria Grazia Di Milia[a], Silvia Bua[b], Claudiu T. Supuran[b,c] and Fabrizio Carta.[b,c*]

[a] Forensic Toxicology Division, Department of Health Sciences, University of Florence, L.go Brambrilla 3, 50134, Florence Italy

[b] Università degli Studi di Firenze, Dipartimento Neurofarba, Sezione di Scienze Farmaceutiche, Polo Scientifico, Via U. Schiff 6, 50019 Sesto Fiorentino, Florence, Italy

[c] Università degli Studi di Firenze, Dipartimento di Chimica, Laboratorio di Chimica Bioinorganica, Rm. 188, Via della Lastruccia 3, 50019 Sesto Fiorentino (Florence), Italy.

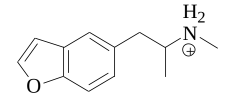

5-MABP ion

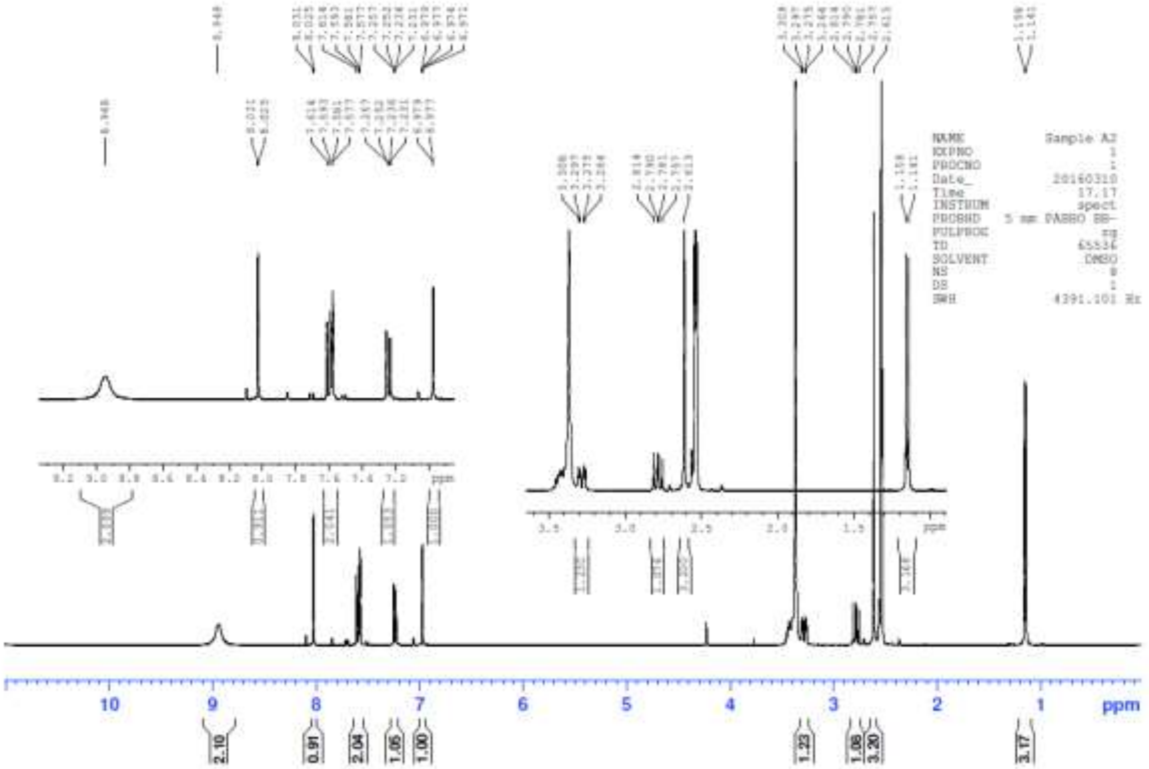

S1. ^{1}H-NMR at 400MHz of 5-MABP ion in DMSO-d_6

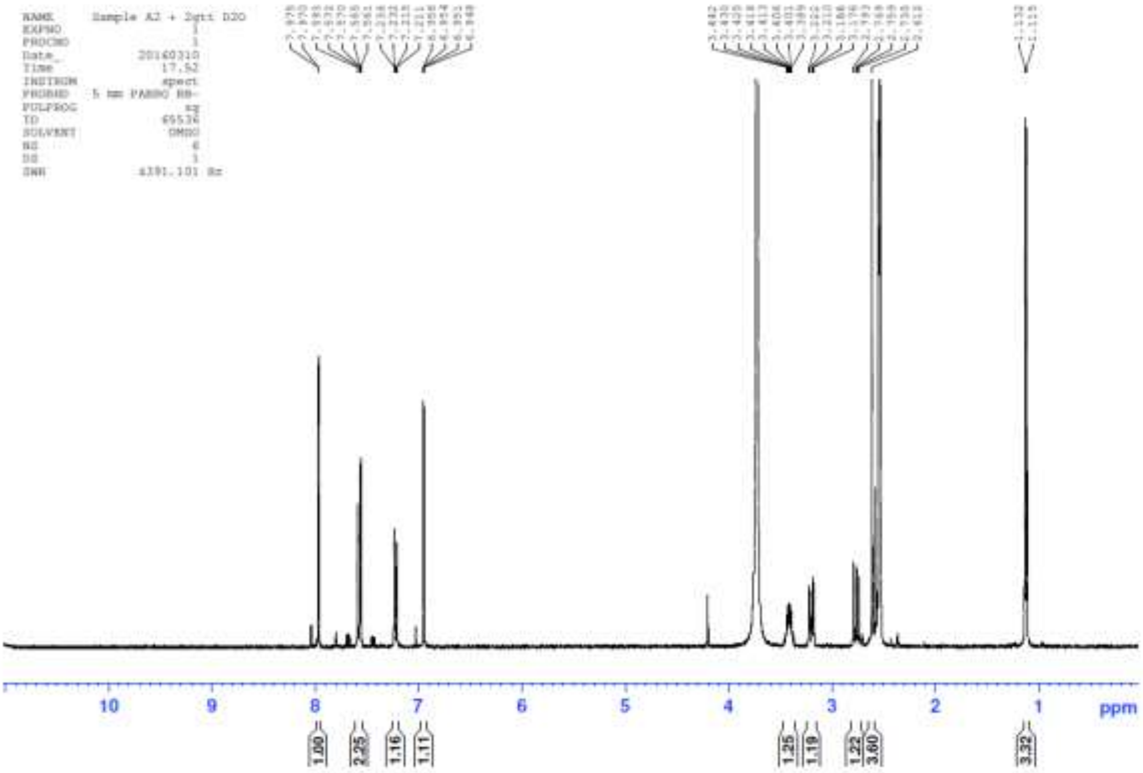

S2. ^{1}H-NMR + D_2O at 400MHz of 5-MABP ion in DMSO-d_6

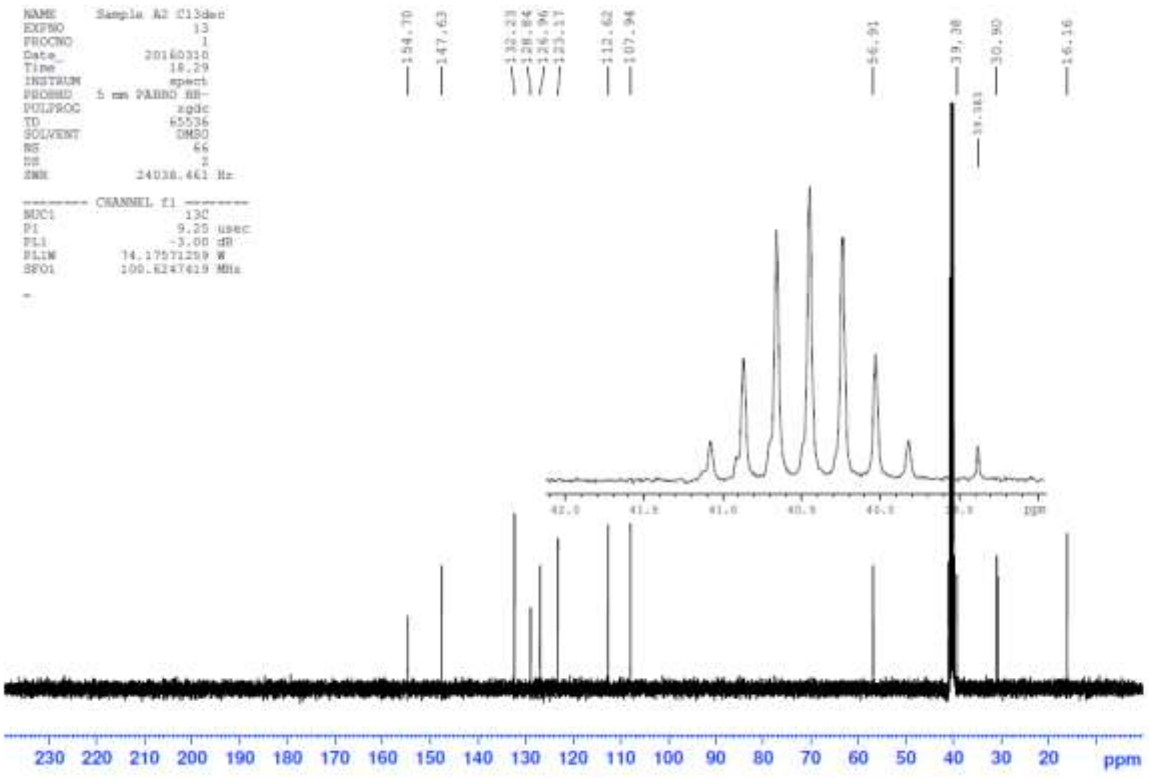

S3. ^{13}C-NMR at 100MHz of 5-MABP ion in DMSO-d_6

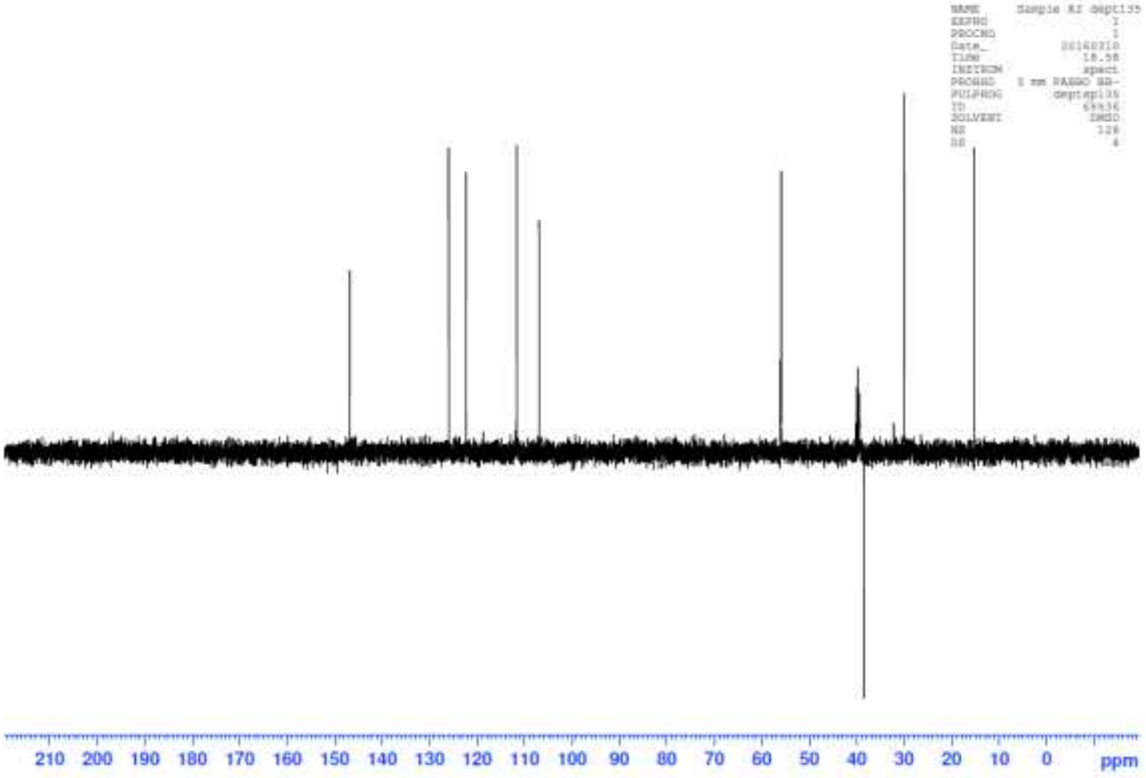

S4. DEPT-135 at 100MHz of 5-MABP ion in DMSO-d_6

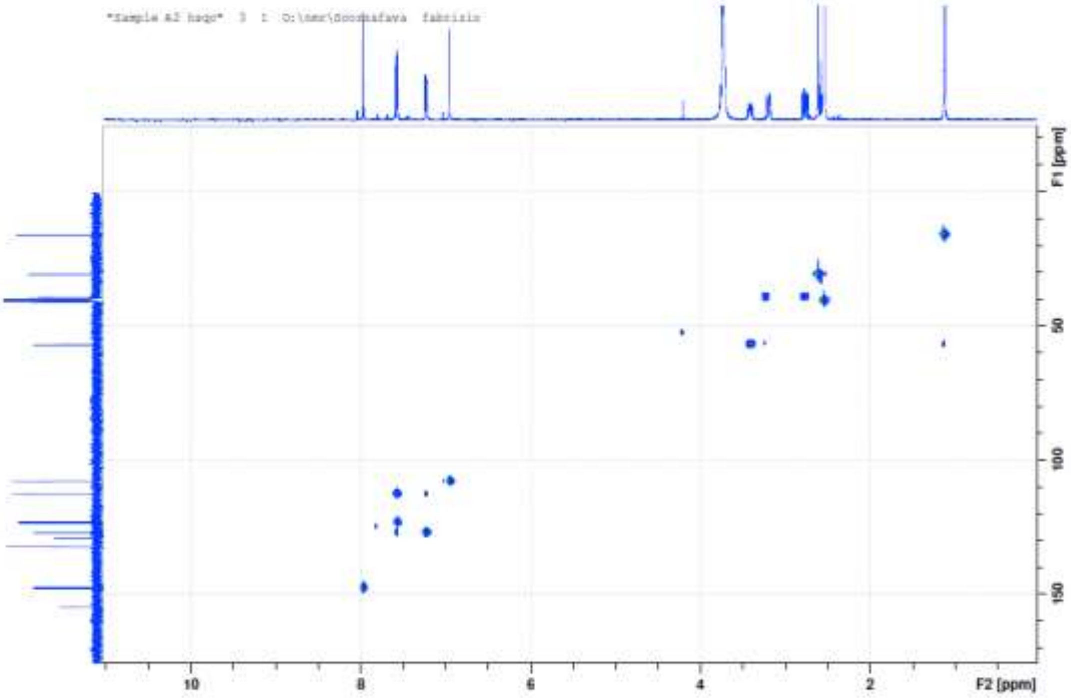

S5. HSQC of 5-MABP ion in DMSO-d_6

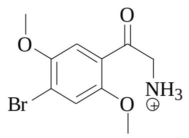

bk-2C-B ion

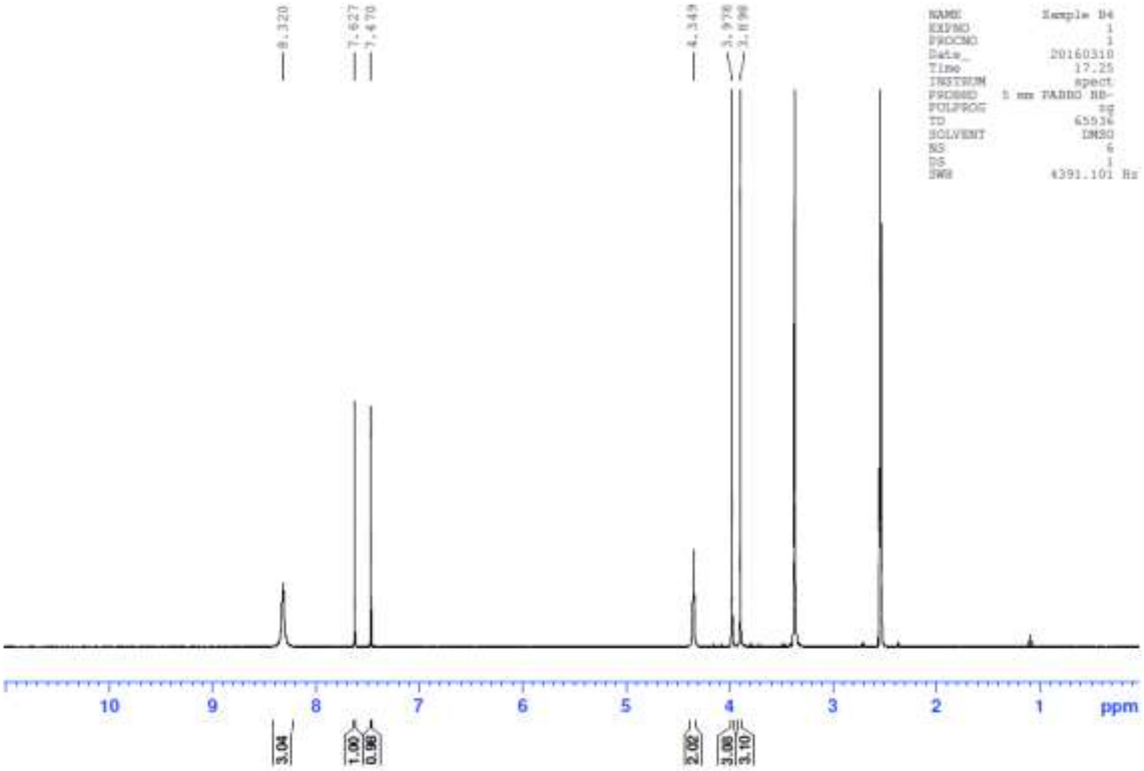

S6. ^{1}H-NMR at 400MHz of bk-2C-B ion in DMSO-d_6

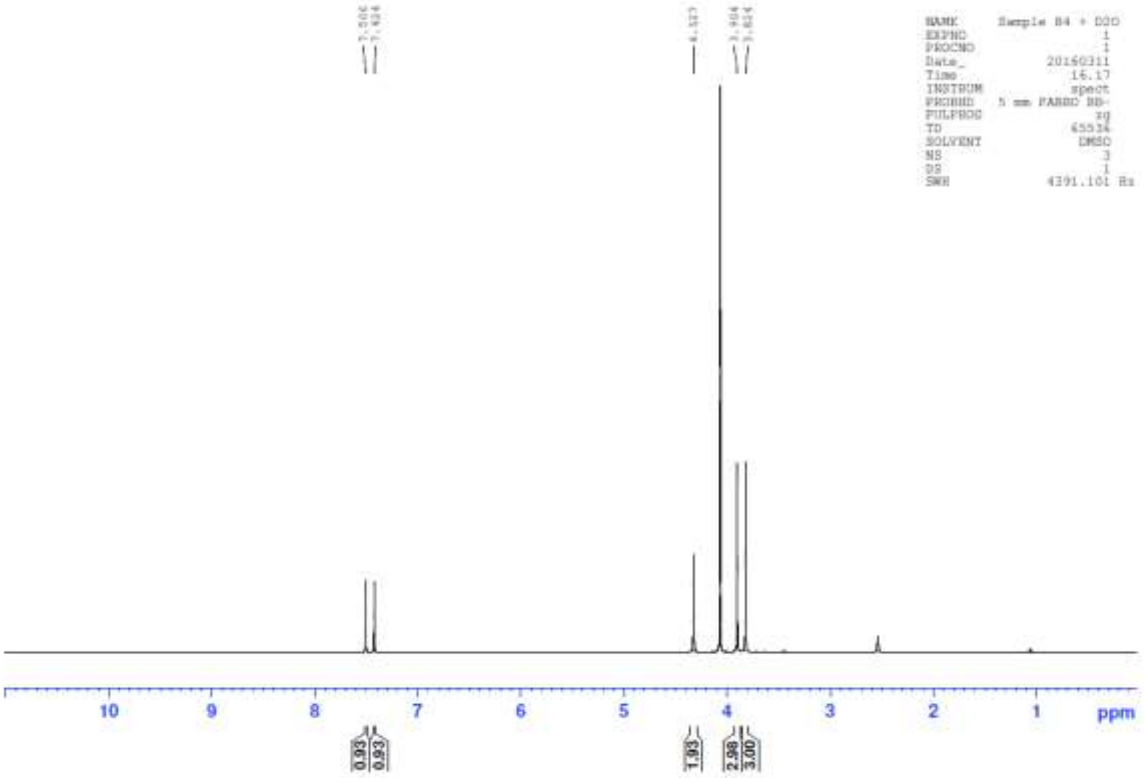

S7. ^{1}H-NMR + D_2O at 400MHz of bk-2C-B ion in DMSO-d_6

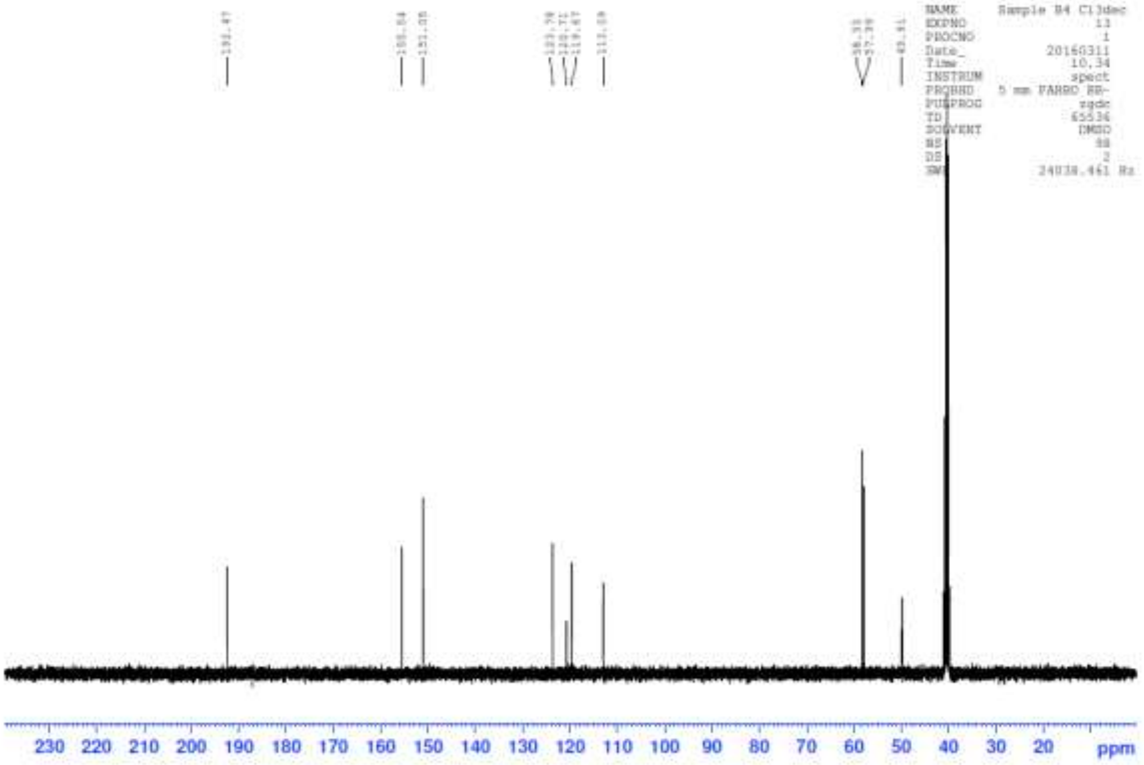

S8. ^{13}C-NMR at 100MHz of bk-2C-B ion in DMSO-d_6

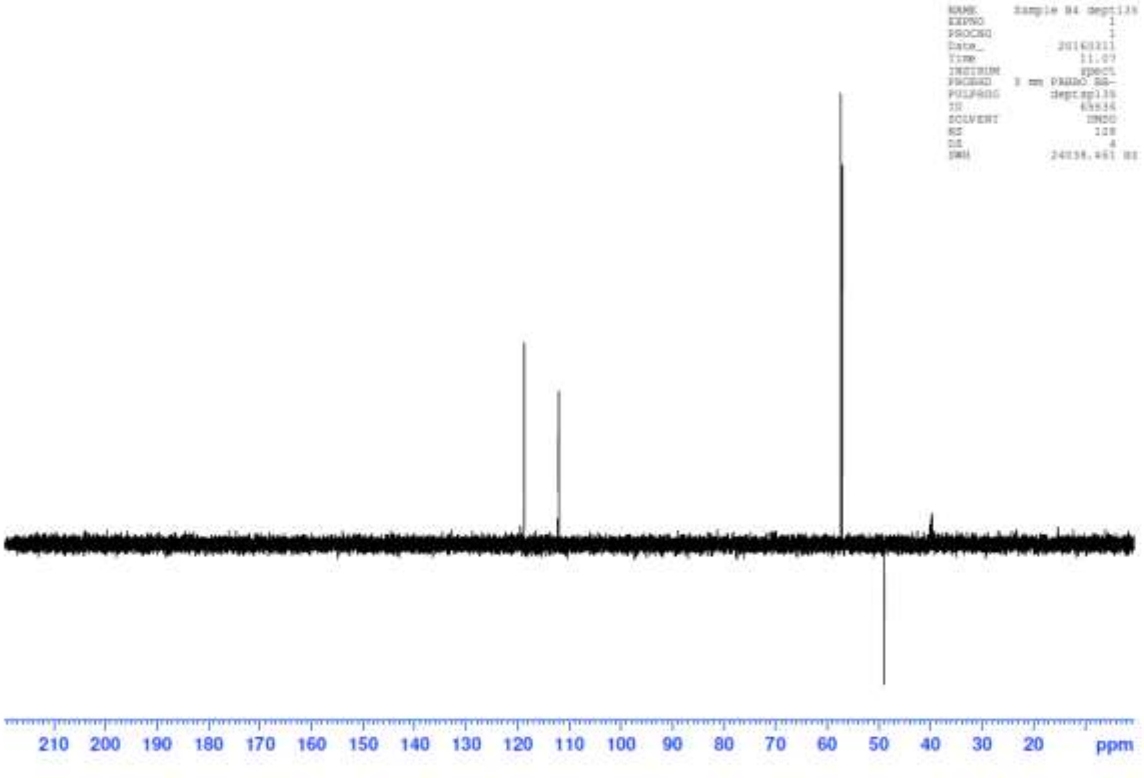

S9. DEPT-135-NMR at 100MHz of bk-2C-B ion in DMSO-d_6

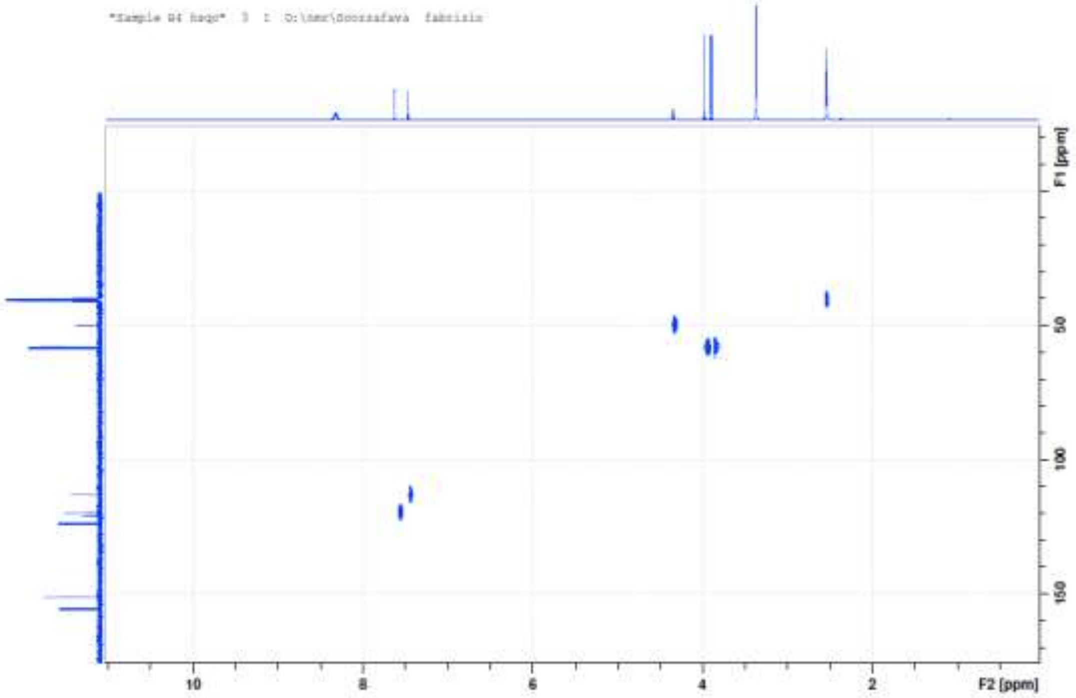

S10. HSQC of bk-2C-B ion in DMSO-d_6
